# Roux-en-Y Gastric Bypass Improved Insulin Resistance via Alteration of the Human Gut Microbiome and Alleviation of Endotoxemia

**DOI:** 10.1155/2021/5554991

**Published:** 2021-07-12

**Authors:** Qiping Shi, Qian Wang, Hui Zhong, Dehai Li, Shuxing Yu, Hengwen Yang, Cunchuan Wang, Zhinan Yin

**Affiliations:** ^1^The First Affiliated Hospital, Jinan University, Guangzhou, China; ^2^Biomedical Translational Research Institute and School of Pharmacy, Jinan University, Guangzhou, China; ^3^Zhuhai Institute of Translational Medicine, Zhuhai People's Hospital Affiliated with Jinan University, Jinan University, Zhuhai, China

## Abstract

**Background:**

Obesity is a main contributing factor for the development of glucose intolerance and type 2 diabetes mellitus (T2D). Roux-en-Y gastric bypass (RYGB) is believed to be one of the most effective treatments to reduce body weight and improve glucose metabolism. In this study, we sought to explore the underlying mechanisms of weight reduction and insulin resistance improvement after RYGB.

**Methods:**

This was a prospective observational study using consecutive samples of 14 obese subjects undergoing bariatric surgery. Main assessments were serum indexes (blood metabolites, glucose-lipid regulating hormones, trimethylamine-N-oxide (TMAO), and lipopolysaccharide-binding protein (LBP), fecal short-chain fatty acids (SCFAs), and gut microbiota. Correlation analysis of the factors changed by RYGB was used to indicate the potential mechanism by which surgery improves insulin resistance.

**Results:**

The subjects showed significant improvement on indices of obesity and insulin resistance and a correlated change of gut microbiota components at 1 month, 3 months, and 6 months post-RYGB operation. In particular, the abundance of a counterobese strain, *Akkemansia muciniphila,* had gradually increased with the postoperative time. Moreover, these changes were negatively correlated to serum levels of LBP and positively correlated to serum TMAO and fecal SCFAs.

**Conclusions:**

Our findings uncovered links between intestinal microbiota alterations, circulating endotoxemia, and insulin resistance. This suggests that the underlying mechanism of protection of the intestine by RYGB in obesity may be through changing the gut microbiota.

## 1. Background

The global trend in mean body mass index (BMI), and the proportion of individuals classified as overweight and obese, is increasing [1], which leads to the prevalence of obesity and its metabolic comorbidities such as type 2 diabetes mellitus (T2D), cardiovascular diseases, and certain cancers.

Roux-en-Y gastric bypass (RYGB) is believed one of the most effective procedures in reducing body weight and improving metabolism [2, 3]. Recent work indicated that RYGB could not only reduce food ingestion, increase gastric emptying, and release satiety-promoting gut hormones [4, 5] but also reduces adipose and serum glucose by altering the structure of the gut microbiota [6]. Additionally, RYGB also significantly improved glucose and insulin responses within subjects that received medication or intensive lifestyle therapy after 5 years, but the exact mechanisms remain unclear [7].

The composition of the microbiota is believed to play a critical role in obesity [8] and diabetes [9]. Gut microbiota could also produce several metabolites and certain potential mediators, such as lipopolysaccharide (LPS) [10] and fecal short-chain fatty acids (SCFAs) [11], to regulate host metabolism. Insulin resistance (IR) is a common feature of both obesity and T2D. It is reported that feeding mice with LPS could enhance adipose tissue inflammation and reduce insulin sensitivity [10], which revealed that gut microbiota may contribute to chronic inflammation, therefore affecting host metabolism. Diet-derived short-chain fatty acid propionate could improve beta-cell function in humans and stimulate insulin secretion from human islets in vitro [12]. However, there are few reports on the association between RYGB and microbiota-related metabolites in obese patients.

Here, we used 16 s rDNA amplicon sequencing to analyze the gut microbiota collected from obesity patients pre- and 1, 3, and 6 months after RYGB. The correlations of certain strains with body weight and fat mass were identified. Moreover, we also detected hormones and microbiota-related metabolites such as SCFAs and inflammation-related proteins as a receptor of LPS, and the correlation of these factors with certain microbiota strains were also discovered.

## 2. Methods

### 2.1. Participants

The study was approved by the Ethics Committee of the First Affiliated Hospital of Jinan University with approval No. [2015] 022. The sponsors had no role in analysis of the data or in the preparation of the manuscript. The first author wrote the initial draft of the manuscript. All authors had independent access to the data and vouch for the completeness and accuracy of the data and for the fidelity of the study to the protocol. A written informed consent was obtained from each participant. All of these subjects were only simply obese and do not have been diagnosed with T2D, but have severe insulin resistance. The patients were from two provinces (Guangdong/Guangxi Province, China) to reduce the effects of geographical and dietary differences on the flora. These patients would undergo RYGB treatment at the First Affiliated Hospital of Jinan University from March 2015 through January 2016, and we screened 14 patients whose clinical data and experimental samples were complete. Patients were excluded if they had undergone other complex abdominal surgeries or previous bariatric surgery or had hypertension, virus hepatitis, colonitis, autoimmune disease, etc.

Participate were provided written informed consent, entered a 6-month tracing period, and underwent repeated physical and laboratory evaluations to evaluate the effect of RYGB. Bariatric procedures were performed laparoscopically by one single surgeon. Participants who were undergoing RYGB were evaluated by a surgical dietitian.

### 2.2. Quantification of Trimethylamine-N-Oxide (TMAO) and Short-Chain Fatty Acids (SCFAs)

Fecal SCFAs were determined by gas chromatography mass spectrometry (GC-MS). TMAO was extracted from serum samples and measured by ultra performance liquid chromatography tandem mass spectrometry (UPLC-MS/MS), see Supplementary materials for specific operation steps.

### 2.3. Human Fecal Sample Collection

14 volunteers were recruited. Human fecal sample from each individual was collected and frozen immediately, then genomic DNA extracted, sequenced, and analyzed was carried out at the same time.

### 2.4. 16S rDNA Gene Sequencing and Processing

Once the DNA samples were received, DNA concentration and sample integrity testing were performed by fluorometer and agarose gel electrophoresis, respectively. To construct the PCR-based library for 16S rDNA amplicon sequencing, V3-V4 dual-index fusion PCR primer cocktail and PCR master mix (NEB phusion high-fidelity PCR master mix) were added to run the PCR. The PCR products were purified with AmpureXp beads (AGENCOURT) to remove the unspecific products. The qualified libraries were sequenced pair end in the MiSeq System with the sequencing strategy PE250. Raw sequences were filtered to eliminate adapters and low quality reads by inhouse procedure, and paired-end reads with overlap were merged with tags [13, 14]. Chimeras were filtered out by using USEARCH v2.4.3 with the SILVA database v12.8 [15]. Tags were clustered into operational taxonomic units (OTU) at 97% sequence similarity by using QIIME v1.9.1 [16, 17]. OTU representative sequences were taxonomically classified using Ribosomal Database Project (RDP) Classifier v.2.2 trained on the SILVA database v12.8 by QIIME v1.9.1 [18–20]. The OTU table was used to calculate the alpha diversity and beta diversity and provide taxonomic profiles. Subsequent statistical analysis was performed in *R* software (v3.5.2).

To investigate microbial diversity and composition, the effective tags of each sample were clustered into an operational taxonomic units (OTU) with a 97% threshold. OTU numbers were used to estimate microbial diversity [16]. Dissimilarity of the microbial community structure was evaluated by nonparametric multivariate analysis of similarity (ANOSIM) and multiresponse permutation procedure (MRPP). Consistent results were obtained by ANOSIM and MRPP using Bray curtis Weighted Unifrac and Unweighted Unifrac distances [21, 22]. Metastat analysis was performed to find OTUs that exhibited significant differences between groups, based on Fisher's exact test and FDR calibration [23]. Indicator species were surveyed to identify OTUs specifically associated with the different groups. An indicator species is a biological species that has a high indicator value for a group of sites, which represent an environmental condition of interest. Changes in indicator species are used to identify changes in ecosystems [24]. Indicator species differ from species associations in that they are indicative of particular groups of sites. Good indicator species should be found mostly in a single group of typology and be present at most of the sites belonging to that group [25, 26]. Indicator species analysis was performed with the “indval” function in the *R* package “labdsv” [27]. Indicator value > 0.40 was chosen as the indicator species in each group. We used permutational multivariate analysis of variance (PERMANOVA) to investigate correlations between clinical indices and changes in the gut microbiome in RYGB patients.

### 2.5. Statistical Analysis

We used mean ± SD to describe parametric data and *t*-test paired to evaluate the difference between each time point effect and one-way ANOVA-repeated measures to assess the effect of RYGB. For nonparametric data, the median with interquartile range was using and Wilcoxon matched pairs signed rank test to evaluate.

Unless otherwise stated, statistical analyses were made in the *R* software. Differential abundances of phyla, genera, and species were tested by Welch two sample *t*-test for comparison between two groups. *P* values were adjusted by the Benjamini-Hochberg correction for multiple tests when required.

## 3. Result

### 3.1. RYGB Changed the Composition of Gut Microbiota

**Figure 1:**
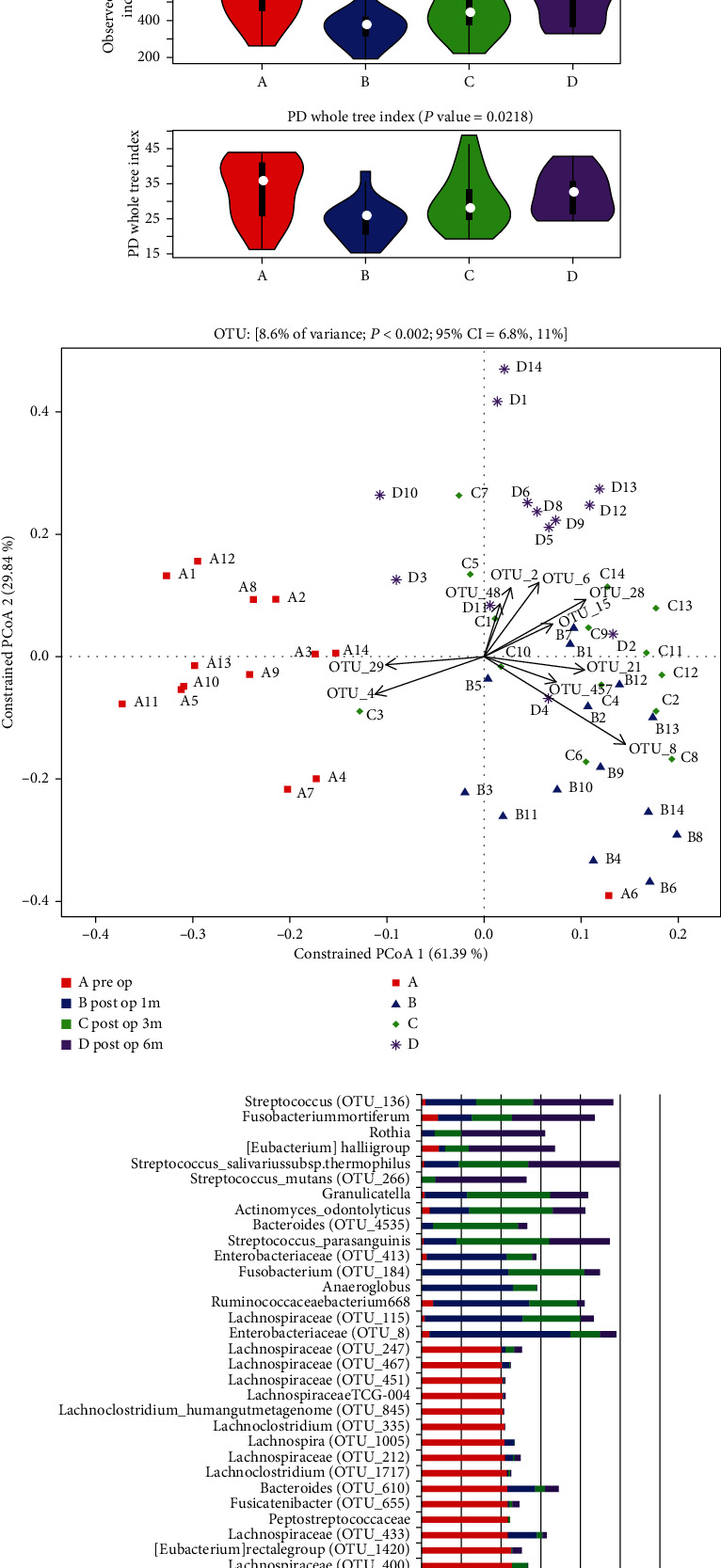
RYGB changed the composition of gut microbiota. Comparison among shotgun sequencing data of stool samples from the preop group, post-1 m-op group, post-3 m-op group, and post-6 m-op group, each group contain 14 patients. (a) The abundance of species in each group. Abundance of species belonging to the preop group was the highest in all the phases, lowest in the post-1 m-op group, recovered gradually, and approached to the preop group at post-6 m-op. (b) Canonical analysis of principal coordinates among 4 groups. There was significant difference in the component of microbiota between the preop group and postop groups and no much diversity among postop groups. (c) Indicator species in each group. Chose OTU over 0.4 as the indicator value when it exists in one group. There were more indicator species in the preop group and more “common” indicator species belonging to three postop groups contemporary.

Then, we research analyzed fecal samples from these participants before and 1, 3, and 6 months after operation to identify whether RYGB could alter gut microbiota composition in the short run. The abundance of species belonging to post-1 m-op group was the lowest in all phases and then gradually recovered. 6 months postoperation, the abundance of species approached similar to the preop group (Figure 1(a)).  Figure 1(b) shows the significant differences in microbiota composition between the preop group and postop groups through canonical analysis of principal coordinates. No differences in the diversity of microbiota among the post-1 m-op group, post-3 m-op group, and post-6 m-op group.

Indicator species were used to distinguish the particular species in pre- and postop groups.  Figure 1(c) shows that there were more indicator species which had stronger specificity in the preop group and more “common” indicator species belonging to the three postop group contemporaries, such as *f_Enterobacteriaceae, f_Lachnospiraceae, f_Ruminococcaceae, f_Streptococcaceae*, and *g_Rothia*.

### 3.2. Associations of Gut Microbial Species with Clinical Parameters

**Figure 2:**
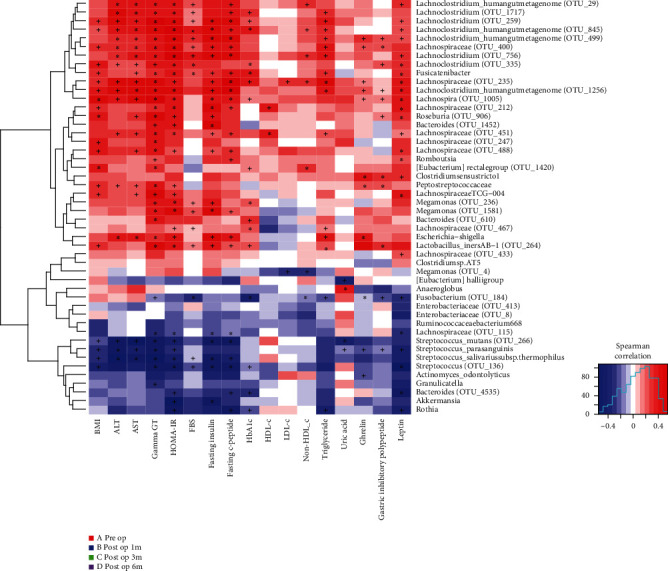
Association of gut microbial species with clinical parameters. Correlation of diversity species in each group and metabolic indices. Most of the diversity species in the preop group had positive correlation while diversity species in postop groups had negative correlation with BMI and other metabolic indices.

To determine whether RYGB changes the gut microbiota in a correlational manner or a coincidental manner, BMI and 12 metabolic indices, including alanine aminotransferase (ALT), aspartate aminotransferase (AST), gamma-glutamyl transpeptidase (gamma-GT), insulin resistance index (HOMA-IR), fasting glucose (FBS), fasting insulin, fasting c-peptide, glycosylated hemoglobin (HbA1c), triglyceride, ghrelin, gastric inhibitory polypeptide (GIP), and leptin, were correlated with alterations in the gut microbiome (Supplemental Table 1, 2). Study selected 48 operational taxonomic units (OTUs) as diversity species belonging to the intersection of indicator species and significant difference species, including 32 OTUs in the preop group, 7 OTUs in the post-1 m-op group, 4 OTUs in the post-3 m-op group, and 4 OTUs in the post-6 m-op group as the diverse species. Most of the diversity species in the preop group had a positive correlation with BMI and metabolic index and that was reversed in the postop group (*P* < 0.05; Supplemental Table 3, 4; Figure 2). What is more, hormones such as circulating GIP and leptin concentration also had a strong negative correlation with diversity species (Figure 2) and decreased following the change of gut microbiota, which indicated that the diversity species may constitute potential biomarkers linking gut microbiota and metabolic status.

### 3.3. RYGB Downregulates the Levels of Lipopolysaccharide-Binding Protein (LBP) in Obese Patients through Altering the Microbiota

**Figure 3:**
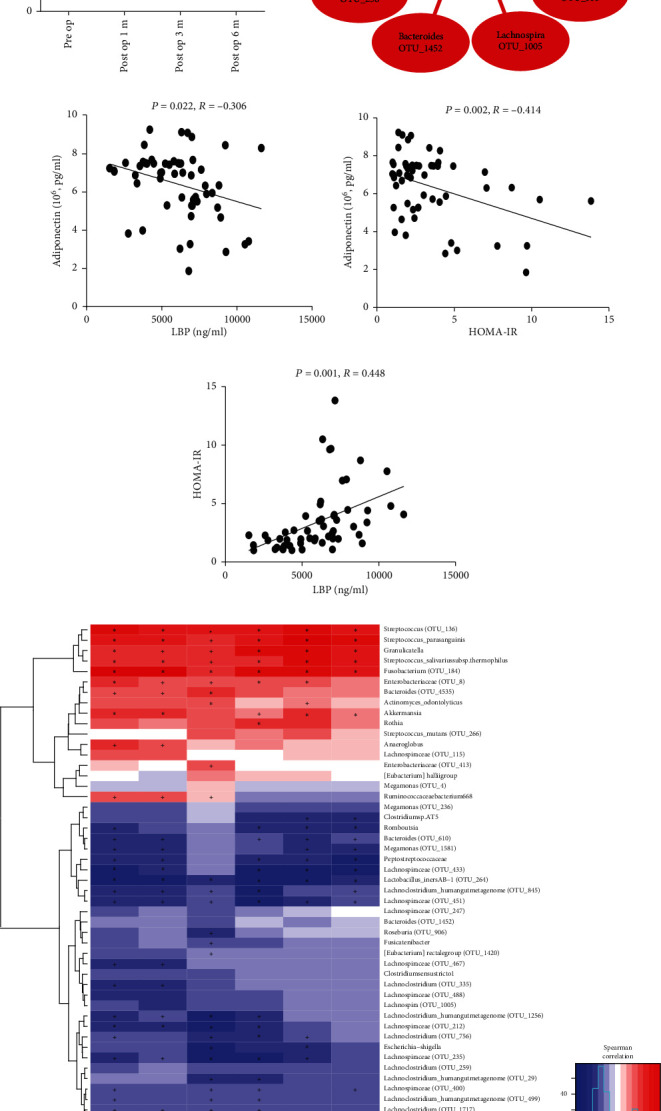
RYGB downregulates the levels of LBP and increases the formation of SCFAs in obese patients. (a) Serum levels of LBP in obesity patients in preop, post-1 m-op, post-3 m-op, and post-6 m-op group. Serum levels of LBP had decreased after RYGB. (b) Correlation network between LBP and diversity species. Red and purple edges denote Spearman's rank correlation (red denotes positive correlation, and purple denotes negative correlation), and difference color circles denoted different groups, respectively. Serum levels of LBP existed a positive correlation with diversity species in the preop group and were negative correlated with postop groups. (c) Correlation between adiponectin and LBP. (d) Correlation between adiponectin and HOMA-IR. (e) Correlation between LBP and HOMA-IR. (f) Correlation network between SCFAs and diversity species. Levels of all these SCFAs in feces had negative correlation with diversity species in the preop group and existed positive correlation in postop groups.

To further understand how RYGB modify gut microbiome and improve IR, serum LBP was measured to reveal the serum level of bacteria product LPS that could be a triggering factor of IR.  Figure 3(a) indicates that after the RYGB operation, the serum level of LBP was decreased significantly over time. The study chose diversity species to analyze the correlation of LBP and microbiota and found that diversity species in the preop group had the positive correlation with the concentration of serum LBP, and diversity species in postop groups had the negative correlation with LBP (Figure 3(b), Supplemental Table 5). What is more, data also showed that LBP was negatively correlated with adiponectin (*P* < 0.05, *R* − 0.306, Figure 3(c)) and so was the relationship of adiponectin and HOMA-IR (*P* < 0.05, *R* = −0.414, Figure 3(d)). In the meantime, LBP showed strong positive correlation with HOMA-IR (*P* < 0.05, *R* = 0.448, Figure 3(e)).

**Figure 4:**
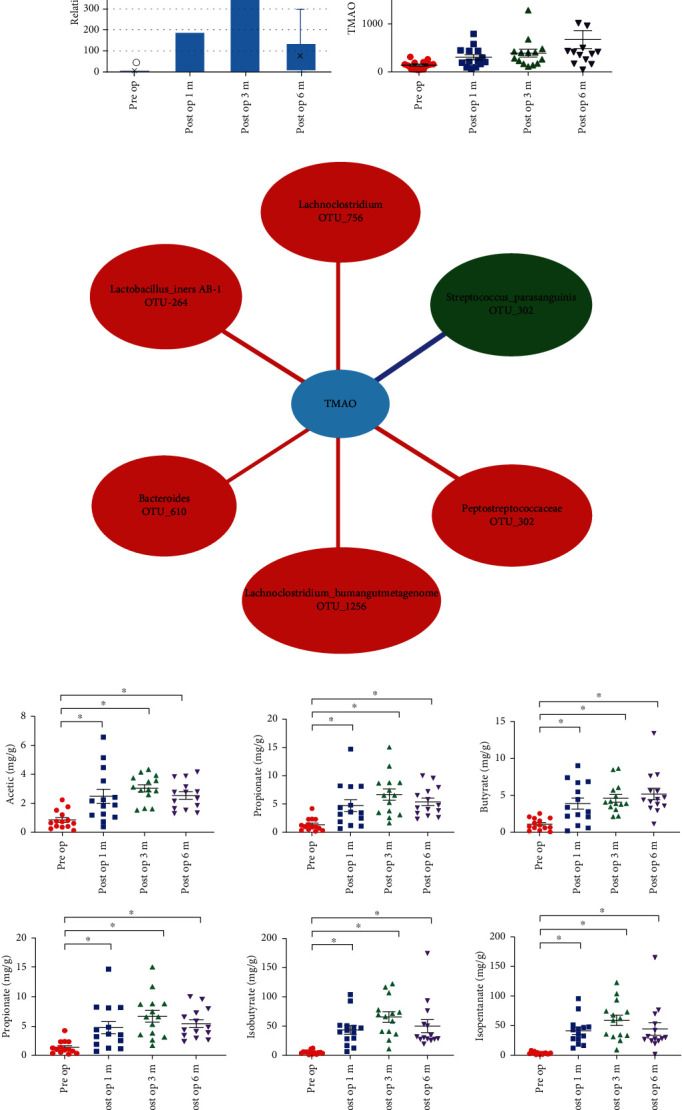
Major increase in microbiota-dependent proatherogenic metabolite TMAO after RYGB. (a) Abundance of *Akkermansia* in obesity patients in preop, post-1 m-op, post-3 m-op, and post-6 m-op group. *Akkermansia* hardly existed in the preop group, recovered after RYGB gradually and stabilized in the post-6 m-op group. (b) Serum levels of TMAO in obese patients in preop, post-1 m-op, post-3 m-op, and post-6 m-op group. Serum levels of TMAO had increased after RYGB. (c) Correlation network between TMAO and diversity species. Red and purple edges denote Spearman's rank correlation (red denotes positive correlation, and purple denotes negative correlation), and difference color circles denoted different groups, respectively. Serum levels of TMAO existed a positive correlation with diversity species in postop groups. (d) Levels of SCFAs in patients' feces in preop, post-1 m-op, post-3 m-op, and post-6 m-op group. Levels of acetate, propionate, butyrate, pentanoate, isobutyrate, and isopentanoate in feces had increased after RYGB. ^∗^From *t-*test-paired or Wilcoxon matched-pairs signed rank test vs. preop group.

*Akkermansia muciniphila* is one mucin-degrading bacterium, and Figure 4(a) also shows that the abundance of *Akkemansia muciniphila* waslower in the preop group and enhanced in the postop group which speculated that the increasing abundance of *Akkemansia muciniphila* may improve the gut barrier integrity through which downregulated the transportation of LPS occurred.

### 3.4. RYGB Increases the Formation of SCFAs

A possible mechanism by which gut microbiota improves the gut barrier integrity is to produce SCFAs. To further identify whether RYGB could change the formation of SCFAs, the levels of SCFAs in patients' faeces were recorded. As expected, acetate, propionate, butyrate, pentanoate, isobutyrate, and isopentanoate levels increased in postop groups (Figure 4(d)). Furthermore, all of these SCFAs had the negative correlation with the diversity species in the preop group and a positive correlation in the postop group (Figure 3(f) and Supplemental Table 6, 7). Results further indicated that RYGB could enhance the formation of SCFAs through which to preserve the gut barrier, improve stability, decline the permeability further to reduce LPS shifting into blood circulation, decreasing LBP, and alleviating endotoxemia.

### 3.5. Microbiota-Dependent Proatherogenic Metabolite TMAO Increased after RYGB

TMAO has been reported to be a microbiota-dependent proatherogenic metabolite, whether RYGB may help to control this metabolite was worth to detect. However, the results found that the TMAO level was significantly increased in a short period (1 month after surgery) and continued to grow over time (Figure 4(b)). Through analyzing the correlation of TMAO with diverse microbiota OTUs in each group, the results indicate that diversity OTUs in the preop group had a negative correlation with the serum level of TMAO, while the postop group had a positive correlation with TMAO (Figure 4(c), Supplemental Table 8).

## 4. Discussion

Study [28] has shown that after the laparoscopic band operation, patients in the early phase of T2D could restore insulin sensitivity within several months after surgery. These dramatic effects suggested rapid insulin sensitizing effects and pancreatic *β*-cell enhancing effects related to intestinal bypass of nutrients, usually occurring prior to maximal weight loss [29]. The results of our follow-up analysis show that 6 months after bariatric surgery sustained weight reduction and insulin resistance remission. Patients who underwent RYGB were capable of achieving a HbA1c level under 6% and HOMA-IR level under 2. Participants would yield lower fat percentage and content when 3 months after RYGB (Supplemental Table 1). Thus, weight loss and insulin sensitivity recovery are the main advantages of the surgery. However, in our results, there was no significant change of LDL-c between post and proop, and we speculated that it may be due to the special high-protein diet that is requested by the doctor after surgery. Fatty liver index (FLI), which has been used as a noninvasive estimate of hepatic steatosis [30], is much lower at 3^rd^ month and 6^th^ month (Supplemental Table 1). It showed that RYGB might alleviate nonalcoholic fatty liver disease and is not fully dependent on serum lipid levels as a recovery indicator.

We also found that the metabolic indices such as BMI, ALT, AST, gamma-GT, FBS, fasting insulin, HOMA-IR, and triglyceride showed a positive correlation with diversity OTUs in the preop group and negative correlation in the postop group. These results suggest that glucose and lipid metabolism may be modulated by gut microbiota species.

LBP, a signal-transducing integral membrane protein that specially combined with LPS [31], has been reported to be a marker of obesity, insulin resistance, and “effective endotoxemia” [32]. We also found that RYGB could downregulate the levels of LBP in obese patients. Combined with our microbiota results, we found serum LBP levels had a positive correlation with diversity OTUs in the preop group and negative in the postop group, which suggested that the levels of LBP in the circulation may reflect the changes of these bacterial species that influence the production of LPS in the intestinal tract.

The uppermost control of the gut barrier relies on an intact epithelium where tight junctions seal the space between individual epithelial cells through which to maintain the epithelial integrity [33]. *Akkermansia muciniphila* stimulated the expression of 2 tight junction proteins, occluding and ZO-1 in intestinal epithelial cells through which the gut barrier was preserved [33, 34], and the number of *Akkermansia muciniphila* was markedly decreased in both genetically and HFD-induced obese mice [34]. Normalizing the proportion of *Akkermansia muciniphila* in obese mice would lead to improvement of several metabolic disorders, including metabolic endotoxemia, fat mass gain, adipose tissue inflammation, and insulin resistance [34, 35]. *Akkermansia muciniphila* could attenuate atherosclerotic lesions by ameliorating metabolic endotoxemia-induced inflammation and restoration of the gut barrier [36]. In our results, we found that *Akkermansia muciniphila* was highly enriched in patients after RYGB and speculated that enrichment of *Akkermansia muciniphila* might attenuate insulin resistance by preservation of the gut barrier and amelioration of endotoxemia-induced inflammation.

Changes in the intestinal flora in SCFA composition have been consequently found to be associated with the development of obesity, insulin resistance, and diabetes [37–39]. Supplementation of butyrate to HFD could increase insulin sensitivity and reduce body weight in C57BL/6 mice [40]. Administration of probiotic VSL#3 to HFD fed mice could increase butyrate production and then suppress body weight gain and insulin resistance [38]. Withal, appropriate intake of dietary fibers was associated with a SCFAs profile that could increase the anti-inflammatory response in the body; however, a HFD was often associated with a reduction of SCFAs and an increase in LPS levels [41]. In our results, we found that not only butyrate but also acetate, propionate, pentanoate, isobutyrate, and isopentanoate were all increased in months 1, 3, and 6 after operation. What is more, diversity OTUs in the preop group had a negative correlation with these SCFAs and had a positive correlation in postop groups. Therefore, we considered that obese participants who underwent RYGB may have had a different structure of microbiota, which could heighten SCFA levels after operation. The increased SCFAs contribute to decreasing the intestinal permeability to some extent, which reduces the translocation of LPS to tissues, thus resulting in the decrease of endotoxemia-induced inflammation and subsequently ameliorated insulin resistance.

We found that TMAO levels were evidently increased in months 1, 3, and 6 after operation. Our result was in line with the recent report that TMAO was not elevated in obese patients or reduced by lifestyle interventions but increased approximately twofold after bariatric surgery [42]. The diversity of OTUs in the preop group existed a negative correlation with serum TMAO levels and a positive correlation in the postop group. It manifested that RYGB may change the composition of microbiota through which to turn up the circulation levels of TMAO. Studies had found that elevated TMAO levels could predict the incidents of thrombotic events in human subjects [43]. Clinical data showed that there is a positive correlation between high blood plasma TMAO levels and increased CVD risk, independent of traditional risk factors, such as kidney failure or diabetes [44]. With the combination of literature and our data, TMAO levels were evidently increased in months 1, 3, and 6, which might predict the high CVD risk after RYGB. On the other hand, a five-year outcome data from a STAMPEDE trial showed that the incidence of CVDs had no obvious increase in patients undergoing RYGB [7]. We speculated that , due to changes in intestinal flora, TMAO may not be used as a standard for predicting CVDs in patients undergoing weight-loss surgery, and it is also needed to be cautious for patients who have any kind of cardiovascular risk to choose RYGB [45].

Nevertheless, we also obtained results that obese patients could have the higher abundance of *Akkermansia muciniphila* and more SCFAs produced by the flora, all of which might improve the integrity of the gut barrier. We inferred that the changes of microbiota component affecting the production of LPS and the decreased intestinal mucosal permeability might be the reason for the reduced translocation of LPS, together with these two synerise attenuated endotoxemia-induced inflammation, then reduce CVD risk. RYGB directly changed the gastrointestinal tract structure of obese individuals through which to affect the intestinal flora and the intestinal immune system indirectly. Nevertheless, the regulating function of the microbiota in the intestinal immunity is not fulfilled by one single germ cell, but carried out by interactions between different strains within the flora. Complete rejection of RYGB benefits would be unreasonable when only considering TMAO as a disease predictor. In the meantime, we also should not exaggerate the benefits of the surgery either.

## 5. Conclusion

Our findings uncovered links between intestinal microbiota alterations, circulating endotoxemia, and insulin resistance, suggesting that the underlying mechanism of the protective effect of RYGB in obesity may be through changing gut microbiota. Since it is only a single-center prospective observational study, the results of the study are limited to a certain extent. We will continue further multicenter prospective studies to evaluate the efficacy of this novel template.

## Data Availability

The 16S rRNA sequencing data were submitted to EBI with accession number PRJEB32163 http://www.ebi.ac.uk/ena/data/view/PRJEB3216.

## Abbreviations

T2D: Type 2 diabetes mellitus
RYGB: Roux-en-Y gastric bypass
TMAO: Trimethylamine-N-oxide
LBP: Lipopolysaccharide-binding protein
SCFAs: Fecal short-chain fatty acids
BMI: Body mass index
TMA: Trimethylamine
CVD: Cardiovascular diseases
GIP: Gastric inhibitory polypeptide
GLP-1: Glucagon-like peptide-1
ALT: Alanine aminotransferase
AST: Aspartate aminotransferase
Gamma-GT: Gamma-glutamyl transpeptidase
TG: Triglycerides
TC: Total cholesterol
HDL-c: High-density lipoprotein cholesterol
LDL-c: Low-density lipoprotein cholesterol
FLI: Fatty liver index
HbA1c: Glycosylated hemoglobin.

## Supplementary Materials

Supplementary 1 Data collection, assessment, and method of quantification trimethylamine-N-oxide (TMAO) and short-chain fatty acid SCFAs could be found in supplemental data; also, the change of metabolic profiles after RYGB was displayed in supplemental.

Supplementary 2 Supplemental Table 1. General characteristics of bariatric surgery patients. Values presented as mean ± SD or median (interquatile). ^∗^From *t*-test-paired vs. preop group or Wilconxon matched-pairs signed rank test. *P* value: from RM one-way ANOVA-repeated measures or Friedman test.

Supplementary 3 Supplemental Table 2. Changes of serum glucose regulating hormone and lipid regulating hormone in patients after bariatric surgery. Values presented as mean ± SD. ^∗^From *t*-test-paired vs. preop group or Wilconxon matched-pairs signed rank test. *P* value: from one-way ANOVA-repeated measures.

Supplementary 4 Supplemental Table 3. Correlation between OTU classification and clinical parameters.

Supplementary 5 Supplemental Table 4. *P* value of OTU classification and clinical parameters.

Supplementary 6 Supplemental Table 5. Correlation between OTU classification and serum LBP concentration.

Supplementary 7 Supplemental Table 6. Correlation between OTU classification and feaces SCFAs concentration.

Supplementary 8 Supplemental Table 7. *P*-value of OTU classification and feaces SCFAs concentration.

Supplementary 9 Supplemental Table 8. Correlation between OTU classification and serum TMAO levels.
