# Supplementary material for: Roux-en-Y Gastric Bypass Improved Insulin Resistance via Alteration of the Human Gut Microbiome and Alleviation of Endotoxemia
Source: Biomed Res Int. 2021 Jul 12;2021:5554991. doi: 10.1155/2021/5554991 (PMC8294027; doi:10.1155/2021/5554991)
Supplement: Supplementary 1 — Data collection, assessment, and method of quantification trimethylamine-N-oxide (TMAO) and short-chain fatty acid SCFAs could be found in supplemental data; also, the change of metabolic profiles after RYGB was displayed in supplemental. [file 5554991.f1.docx]

**Supplemental data**

**Methods**

**Data collection and assessment**

We assessed body weight, blood pressure, and collected blood samples for clinical biaochemical analyses after an overnight fast for at least 10 hours. The sera were separated from the whole blood. The samples were put in vials and store at -80℃ for later analysis. Fasting glucose, serum alanine aminotransferase (ALT), aspartate aminotransferase (AST), and gamma-glutamyl transpeptidase (gamma-GT), creatinine, uric acid, lipid profile, including triglycerides (TG), total cholesterol (TC), high-density lipoprotein cholesterol (HDL-c), and low-density lipoprotein cholesterol (LDL-c) were measured using an autoanalyser (Beckman Coulter AU5800). Fasting serum insulin was measured using a double antibody radioimmunoassay (DSL, Webster). Insulin resistance index (HOMA-IR) was calculated using homeostasis model assessment methods, defined as fasting insulin (IU/ml) × fasting glucose (mmol/l)/22.5. HbA1c was measured by high-pressure liquid chromatography. All these index will be analyses at baseline and at months 1, 3 and 6.

Most of the serum metabolic hormone was analyzed using Luminex multicytokines panel according to the manufacture’s instruction. LBP and adiponectin were detected using Elisa kits (BioLegend). The samples were brought to room temperature according to the respective manufacture’s instruction.

**Quantification trimethylamine-N-oxide (TMAO) and short-chain fatty acids SCFAs**

Fecal SCFAs were determined by gas chromatography-mass spectrometry (GC-MS). TMAO was extracted from serum samples and measured by ultra-performance liquid chromatography-tandem mass spectrometry (UPLC-MS/MS).

GC-MS was used to measure SCFAs of fecal samples at baseline and at months 1, 3 and 6 fecal samples. Take 400mg for each sample in the 2ml EP tubes, extracted with 1.6ml ddH_2_O, vortex for 30s; then 5000rpm centrifuged for 20 min, at 4°C. Carefully took the supernatant 0.8ml; then added 0.2ml 50% H_2_SO_4_ and 1ml internal standard liquid (50μg/ml in aether). Vortex for 30s before centrifuged at 12000rpm for 10 min, at 4°C. Incubate for 30min in 4℃, take the supernatant (about 0.5ml) for GC analysis. GC analysis was performed using an SHIMADZU GC-2010 gas chromatograph system c. The system utilized a DB-FFAP column (30m×250μm inner diameter, 0.25μm film thickness; Agilent, USA). A 1μl aliquot of the analyte was injected split mode (split ratio is 5:1). Helium was used as the carrier gas, the gas flow rate through the column was 2ml min−1. The initial temperature was kept at 100°C for 1 min, then raised to 150°C at a rate of 5°C min−1, then raised to 240°C at a rate of 50°C min−1, then kept for 17.2min at 240°C.The FID temperatures was 300°C. The gas flow rate of tail gas, hydrogen and air were 30ml min−1, 40ml min−1, 400ml min−1.

TMAO was extracted from 100μl of plasma also at baseline and at months 1, 3 and 6. Prepared 100μg/ml of the standard solution, using MassHunter Optimizer software, set the type of Sample introduction; optimized fragmentor range; Collision Energy optimization scope; data collection instruments method (Acq Method); Ion Source,and Polarity, automatically optimize the standard optimum parameters. Weight 100μl sample, transfer into 2ml EP tubes. Add 900μl Methanol:ACN:H_2_O（2:2:1）, vortex for 30 s. Ultrasonic extraction 15 minutes at 4℃，incubated at -20 ℃ for 1 hour. Centrifuge for 15 min at 12000rpm, 4℃; remove supernatant into disposable syringe filter plug with 0.22μm pore size filter inside Test samples on 6400 series triple quadrupole LC/MS sys.

**Result**

**Metabolic profiles are improved and glucose-lipid regulating hormones are ameliorated after RYGB**

In our study, individuals who were obese with IR but were not diabetic were generally younger. The main clinical and biochemical characteristics of the study groups are shown in Supplemental Table 1. Study chose 14 obese patients (6 male, 8 female; body mass index (BMI), 37.70±6.77 kg/m^2^; abdominal circumference, 122.5±18.06 cm; age, 29.07±8.75 years; data are mean±s.d.), and fasting glucose, 5.62mmol/l (4.9 mmol/l, 6.22 mmol/l); HOMA-IR, 4.65 (2.61, 9.69); glycosylated hemoglobin (HbA1c), 5.95% (5.1%, 6.4%); data are median (quartiles)). Briefly, BMI, abdominal circumference，fasting insulin, HOMA-IR, total cholesterol (TC), alanine aminotransferase (ALT) andaspartate aminotransferase (AST) were decreased at post- 6m-op (6 months after operation) group. Fat content, fat percentage and especially fatty liver index（FLI) [1] were all significantly decreased, though low-density lipoprotein cholesterol (LDL-c), high-density lipoprotein cholesterol (HDL-c), non-HDL-c, TC, HbA1c and 1,25-Dihydroxyvitamin D3 did not have significant changes at post-op groups when compared with pre-op group (Supplemental Table 1). These results indicated that RYGB could not only decrease the weight, fat content and percentage of the obese patients, but also significantly improve fatty liver.

Hormones that were regulated by glucose and lipid were analyzed and found that glucose-dependent gastric inhibitory polypeptide (GIP) was slightly decreased after operation (*P*<0.05, Supplemental Table 2). In this study, serum leptin levels were also significantly decreased after RYGB (*P*<0.05, Wilcoxon mathed-pairs signed rank test, Supplemental Table 2), suggesting that the leptin resistance in obese patients might be relieved after operation. There was no significant difference of hormones such as glucose-dependent glucagon-like peptide-1 (GLP-1), ghrelin, visfatin, and resistin, between pre-op and post-op groups. What’s more interesting, the serum level of adiponectin, which could effectively improve insulin sensitive, was significantly increased as early as 1 month after RYGB then stayed there in 3 and 6 month after operation (*P*<0.05, Wilcoxon mathed-pairs signed rank test, Supplemental Table 2). Data demonstrated that RYGB could improve metabolism-related endocrine reactions even in a very short time.

**Reference**

1. Bedogni G, Bellentani S, Miglioli L, Masutti F, Passalacqua M, Castiglione A and Tiribelli C. The Fatty Liver Index: a simple and accurate predictor of hepatic steatosis in the general population. BMC Gastroenterol 2006; 6: 33.
