# Supplementary material for: Roux-en-Y Gastric Bypass Improved Insulin Resistance via Alteration of the Human Gut Microbiome and Alleviation of Endotoxemia
Source: Biomed Res Int. 2021 Jul 12;2021:5554991. doi: 10.1155/2021/5554991 (PMC8294027; doi:10.1155/2021/5554991)
Supplement: Supplementary 2 — Supplemental Table 1. General characteristics of bariatric surgery patients. Values presented as mean ± SD or median (interquatile). ∗From t-test-paired vs. preop group or Wilconxon matched-pairs signed rank test. P value: from RM one-way ANOVA-repeated measures or Friedman test. [file 5554991.f2.docx]

| **table 1. General characteristics of bariatric surgery patients** | | |  |  |  |  |
| --- | --- | --- | --- | --- | --- | --- |
| 列1 | **Pre-OP** | **Post-1M-OP** | **Post-3M-OP** | **Post-6M-OP** | ***P* value** |  |
| **Abdominal circumference (cm)** | 122.5±18.06 | 110.93±12.23 | 103.86±12.81* | 97.79±15.08* | 0.000 |  |
| **Body Mass Index (kg/m^2^)** | 37.70±6.77 | 33.35±5.83 | 30.19±5.00* | 27.60±4.38* | 0.000 |  |
| **Fasting glucose(mmol/L)** | 5.62（4.9, 6.22) | 5.44 (5.12, 5.74) | 5.38 (5.03, 5.84) | 5.07 (4.68, 5.55) | 0.384 |  |
| **c-peptide (ng/mL)** | 3.41 (2.72, 4.10) | 2.24 (1.86, 3.36)* | 2.07(1.79, 3.96) | 1.69 (1.2,2.23)* | 0.003 |  |
| **Insulin (mIU/L)** | 17.8 (12.63, 22) | 11.85(7.75, 15.35)* | 9.8(6.85, 16.05)* | 6.4(5.4, 8.95)* | 0.000 |  |
| **HbA1C (%)** | 5.95 (5.1, 6.4) | 5.65 (5, 6.33) | 5.3 (5.08, 5.63) | 5.5 (5.1, 6.2) | 0.226 |  |
| **1,25-Dihydroxyvitamin D3 (ng/mL)** | 19.22±5.98 | 20.72±4.38 | 20.50±7.38 | 20.34±6.40 | 0.911 |  |
| **Low Density Lipoprotein cholesterol(mmol/L)** | 3.21±0.64 | 2.81±0.57 | 3.17±0.73 | 3.00±0.56 | 0.342 |  |
| **High Density Lipoprotein cholesterol(mmol/L)** | 1.11±0.30 | 1.01±0.21 | 1.06±0.17 | 1.17±0.23 | 0.294 |  |
| **Total cholesterol(mmol/L)** | 5.01±0.65 | 4.38+0.79 | 4.76±0.73 | 4.79±0.54 | 0.120 |  |
| **Triglycerides(mg/dL)** | 114.31 (104.43, 259.75) | 107.97 (68.15, 157.31) | 97.79 (72.57, 116.16)* | 70.8 (61.73,102.66)* | 0.022 |  |
| **ALT(IU/L)** | 53.5 (28, 78.3) | 38.5 (27.8, 48) | 20.5 (18.8, 28.8)* | 19 (12.8, 24.5)* | 0.000 |  |
| **AST(IU/L)** | 29.5 (23.3, 44) | 29 (24.5, 34.3) | 20 (18.8, 24.5)* | 16.5 (13.8, 21.3)* | 0.000 |  |
| **γ-GT(IU/L)** | 42.5 （26.8, 71.5） | 21 (16, 27.5)* | 14 (12, 19.3)* | 11.5 (11,15.3)* | 0.000 |  |
| **Uric acid (µmol//L)** | 436.00±81.96 | 510.86±123.74 | 405.00±74.33 | 396.79±76.62 | 0.007 |  |
| **FLI** | 90.24±13.76 | 73±27.78* | 57.35±27.69* | 38.21±27.17* | 0.000 |  |
| **Fat content** | 58092±12590 |  | 49192±11566 |  | 0.620 |  |
| **Percentage of fat （%）** | 44.1±5.59 |  | 39.71±6.96 |  | 0.780 |  |
| **Values presented as mean±SD or median (interquatile)** | | |  |  |  |  |
| *** ：from *t* test-paired *vs.* Pre-OP group or Wilconxon matched-pairs signed rank test.** | | | | |  |  |
| ***P* value ：from RM One-way ANOVA-repeated measures or Firedman test.** | | | |  |  |  |
|  |  |  |  |  |  |  |
