# Supplementary material for: Roux-en-Y Gastric Bypass Improved Insulin Resistance via Alteration of the Human Gut Microbiome and Alleviation of Endotoxemia
Source: Biomed Res Int. 2021 Jul 12;2021:5554991. doi: 10.1155/2021/5554991 (PMC8294027; doi:10.1155/2021/5554991)
Supplement: Supplementary 3 — Supplemental Table 2. Changes of serum glucose regulating hormone and lipid regulating hormone in patients after bariatric surgery. Values presented as mean ± SD. ∗From t-test-paired vs. preop group or Wilconxon matched-pairs signed rank test. P value: from one-way ANOVA-repeated measures. [file 5554991.f3.docx]

**table 2. Changes of serum glucose regulating hormone and lipid regulating hormone in patients after bariatric surgery**

|  | **Pre-OP** | **Post-1M-OP** | **Post-3M-OP** | **Post-6M-OP** | | ***P* value** |
| --- | --- | --- | --- | --- | --- | --- |
| **Ghrelin (pg/ml)** | 759.71±505.95 | 422.40±156.83^*^ | 513.81±196.00^*^ | | 506.41±225.49^*^ | 0.022 |
| **Gastric Inhibitory Polypeptide (pg/ml)** | 677.20±666.97 | 253.43±229.31^*^ | 218.38±143.81^*^ | | 317.63±188.70^*^ | 0.028 |
| **Glucagon-like peptide-1 (pg/ml)** | 252.1±205.59 | 171.49±74.78 | 252.14±205.59 | | 179.42±46.13 | 0.142 |
| **Glucagon (pg/ml)** | 460.11±431.84 | 330.12±42.71 | 343.68±30.26 | | 357.2870.62 | 0.329 |
| **Leptin (pg/ml)** | 24715.19±20303.61 | 7095.29±5962.58^*^ | 5933.03±4943.59^*^ | | 7579.94±8154.43^*^ | 0.001 |
| **PAI (pg/ml)** | 16991.09±13908.26 | 17785.96±14703.61 | 16664.87±13728.99 | | 18081.60±13654.75 | 0.414 |
| **Resistin (pg/ml)** | 1731.45±917.64 | 3139.42±4968.82 | 1880.90±679.9 | | 2323.03±1686.15 | 0.315 |
| **Visfatin (pg/ml)** | 4698.32±3216.06 | 4085.43±4373.00 | 3940.98±2689.46 | | 4246.24±3173.45 | 0.835 |

Values presented as mean±SD.

* ：from t test-paired vs. Pre-op group or Wilconxon matched-pairs signed rank test.

*P* value ：from One-way ANOVA-repeated measures.
