# Supplementary material for: Roux-en-Y Gastric Bypass Improved Insulin Resistance via Alteration of the Human Gut Microbiome and Alleviation of Endotoxemia
Source: Biomed Res Int. 2021 Jul 12;2021:5554991. doi: 10.1155/2021/5554991 (PMC8294027; doi:10.1155/2021/5554991)
Supplement: Supplementary 4 — Supplemental Table 3. Correlation between OTU classification and clinical parameters. [file 5554991.f4.docx]

**Table 3. Correlation between OTU classification and clinical parameters**

| **tax** | **BMI** | **ALT** | **AST** | **Gamma GT** | **HOMA**  **-IR** | **FBS** | **Fasting Insulin** | **Fasting c-peptide** | **HbA1c** | **HDL-c** | **LDL-c** | **non-**  **HDL_c** | **Tri-**  **glyceride** | **Uric**  **acid** | **Ghrelin** | **Gastric**  **Inhibitory**  **Polypeptide** | **Leptin** |
| --- | --- | --- | --- | --- | --- | --- | --- | --- | --- | --- | --- | --- | --- | --- | --- | --- | --- |
| OTU_4 | 0.086 | -0.074 | -0.009 | 0.179 | 0.233 | 0.028 | 0.222 | -0.073 | -0.047 | -0.230 | -0.322 | -0.283 | -0.160 | 0.137 | 0.007 | 0.057 | -0.079 |
| OTU_8 | 0.043 | -0.062 | -0.017 | 0.005 | -0.238 | -0.092 | -0.218 | -0.200 | -0.161 | -0.109 | -0.190 | -0.268 | -0.064 | 0.189 | -0.081 | 0.029 | -0.371 |
| OTU_18 | -0.179 | -0.205 | -0.140 | -0.210 | -0.482 | -0.269 | -0.437 | -0.323 | -0.079 | -0.051 | -0.022 | -0.052 | -0.263 | 0.039 | -0.250 | -0.137 | -0.319 |
| OTU_28 | -0.318 | -0.483 | -0.457 | -0.553 | -0.386 | -0.078 | -0.406 | -0.340 | -0.070 | 0.129 | -0.010 | -0.080 | -0.297 | -0.190 | -0.266 | -0.249 | -0.389 |
| OTU_29 | 0.236 | 0.369 | 0.403 | 0.356 | 0.399 | 0.206 | 0.371 | 0.352 | 0.250 | 0.101 | 0.184 | 0.272 | 0.253 | 0.104 | 0.131 | -0.011 | 0.325 |
| OTU_129 | 0.098 | 0.059 | -0.008 | 0.124 | 0.148 | 0.021 | 0.162 | 0.105 | 0.025 | 0.014 | 0.024 | 0.062 | 0.008 | -0.017 | -0.157 | -0.139 | 0.169 |
| OTU_302 | -0.267 | -0.437 | -0.378 | -0.470 | -0.290 | -0.079 | -0.296 | -0.276 | -0.065 | 0.013 | -0.039 | -0.102 | -0.303 | -0.133 | -0.188 | -0.145 | -0.355 |
| OTU_83 | 0.171 | -0.176 | -0.225 | -0.160 | -0.190 | -0.149 | -0.233 | -0.267 | 0.157 | 0.037 | 0.036 | 0.129 | -0.103 | -0.345 | 0.044 | 0.015 | -0.012 |
| OTU_115 | -0.276 | -0.102 | -0.037 | -0.278 | -0.244 | 0.031 | -0.243 | -0.177 | -0.239 | -0.031 | -0.150 | -0.197 | -0.204 | 0.075 | -0.083 | 0.048 | -0.328 |
| OTU_116 | -0.315 | -0.044 | 0.023 | -0.265 | -0.199 | -0.123 | -0.196 | -0.131 | -0.138 | -0.126 | -0.123 | -0.253 | -0.168 | 0.122 | -0.064 | 0.007 | -0.239 |
| OTU_184 | 0.129 | 0.044 | 0.168 | -0.147 | -0.206 | -0.271 | -0.131 | -0.183 | -0.388 | -0.105 | -0.061 | -0.144 | -0.189 | 0.241 | -0.110 | -0.185 | -0.300 |
| OTU_136 | -0.336 | -0.444 | -0.355 | -0.418 | -0.461 | -0.236 | -0.461 | -0.466 | -0.147 | -0.237 | -0.046 | -0.187 | -0.342 | 0.119 | -0.213 | -0.182 | -0.510 |
| OTU_114 | 0.186 | 0.228 | 0.113 | 0.239 | 0.271 | -0.067 | 0.285 | 0.265 | 0.115 | 0.062 | 0.184 | 0.172 | 0.121 | -0.015 | 0.046 | 0.102 | 0.200 |
| OTU_171 | -0.113 | -0.221 | -0.165 | -0.265 | -0.236 | -0.082 | -0.234 | -0.198 | -0.218 | -0.059 | 0.033 | -0.096 | -0.229 | 0.126 | -0.129 | -0.141 | -0.350 |
| OTU_756 | 0.193 | 0.320 | 0.356 | 0.364 | 0.480 | 0.337 | 0.424 | 0.424 | 0.287 | 0.070 | 0.186 | 0.311 | 0.358 | 0.130 | 0.231 | 0.100 | 0.331 |
| OTU_138 | 0.250 | 0.236 | 0.147 | 0.166 | 0.270 | 0.002 | 0.266 | 0.233 | 0.048 | 0.156 | 0.066 | 0.126 | 0.123 | 0.119 | 0.361 | 0.299 | 0.297 |
| OTU_247 | 0.377 | 0.188 | 0.207 | 0.386 | 0.269 | -0.020 | 0.268 | 0.145 | -0.019 | 0.243 | 0.010 | -0.011 | 0.126 | 0.299 | -0.059 | -0.002 | 0.262 |
| OTU_212 | 0.297 | 0.212 | 0.231 | 0.309 | 0.398 | 0.072 | 0.408 | 0.316 | -0.021 | 0.337 | 0.158 | 0.110 | 0.134 | 0.233 | 0.155 | 0.056 | 0.380 |
| OTU_170 | -0.210 | -0.247 | -0.160 | -0.334 | -0.330 | -0.069 | -0.360 | -0.357 | -0.038 | -0.189 | 0.252 | 0.168 | -0.239 | -0.002 | -0.199 | -0.152 | -0.317 |
| OTU_1452 | 0.304 | 0.231 | 0.221 | 0.334 | 0.404 | -0.030 | 0.432 | 0.271 | -0.112 | 0.103 | 0.105 | 0.062 | 0.006 | 0.126 | 0.053 | -0.040 | 0.258 |
| OTU_1005 | 0.473 | 0.388 | 0.410 | 0.481 | 0.364 | 0.057 | 0.377 | 0.350 | 0.175 | 0.120 | 0.244 | 0.206 | 0.302 | 0.296 | 0.128 | 0.176 | 0.345 |
| OTU_906 | 0.390 | 0.205 | 0.275 | 0.441 | 0.360 | 0.077 | 0.337 | 0.290 | 0.027 | 0.156 | 0.104 | 0.073 | 0.240 | 0.159 | 0.049 | 0.136 | 0.291 |
| OTU_263 | 0.206 | 0.360 | 0.335 | 0.305 | 0.342 | 0.069 | 0.407 | 0.359 | 0.113 | -0.096 | -0.049 | 0.010 | 0.379 | 0.075 | 0.391 | 0.178 | 0.234 |
| OTU_433 | 0.165 | 0.183 | 0.081 | 0.301 | 0.144 | -0.012 | 0.162 | 0.118 | 0.051 | 0.032 | -0.008 | 0.061 | 0.069 | 0.062 | -0.084 | 0.024 | 0.253 |
| OTU_236 | 0.224 | 0.254 | 0.192 | 0.354 | 0.365 | 0.163 | 0.336 | 0.160 | 0.227 | 0.016 | -0.088 | -0.019 | 0.041 | 0.041 | -0.100 | 0.041 | 0.294 |
| OTU_259 | 0.304 | 0.411 | 0.375 | 0.481 | 0.438 | 0.143 | 0.423 | 0.448 | 0.237 | 0.048 | 0.049 | 0.191 | 0.356 | 0.231 | 0.046 | 0.010 | 0.228 |
| OTU_235 | 0.313 | 0.419 | 0.425 | 0.414 | 0.402 | 0.209 | 0.371 | 0.499 | 0.334 | 0.141 | 0.309 | 0.308 | 0.444 | 0.235 | 0.130 | 0.060 | 0.389 |
| OTU_413 | -0.061 | -0.184 | -0.188 | -0.022 | -0.151 | 0.067 | -0.150 | -0.106 | -0.001 | -0.102 | -0.149 | -0.210 | -0.077 | 0.004 | -0.046 | 0.091 | -0.303 |
| OTU_510 | 0.297 | 0.190 | 0.241 | 0.307 | 0.364 | 0.230 | 0.314 | 0.343 | 0.356 | 0.045 | 0.029 | 0.170 | 0.304 | -0.074 | 0.084 | -0.014 | 0.325 |
| OTU_1256 | 0.353 | 0.437 | 0.471 | 0.416 | 0.474 | 0.290 | 0.438 | 0.520 | 0.184 | 0.208 | 0.156 | 0.174 | 0.405 | 0.226 | 0.194 | 0.094 | 0.357 |
| OTU_303 | -0.050 | -0.298 | -0.197 | -0.250 | -0.417 | -0.294 | -0.394 | -0.437 | -0.227 | 0.037 | 0.091 | 0.006 | -0.339 | -0.065 | -0.038 | -0.239 | -0.375 |
| OTU_499 | 0.227 | 0.319 | 0.313 | 0.405 | 0.477 | 0.324 | 0.452 | 0.425 | 0.202 | 0.025 | 0.075 | 0.195 | 0.323 | 0.123 | 0.271 | 0.221 | 0.239 |
| OTU_4535 | -0.247 | -0.175 | -0.124 | -0.203 | -0.297 | -0.195 | -0.271 | -0.301 | -0.327 | 0.017 | -0.016 | -0.088 | -0.268 | -0.026 | 0.071 | -0.089 | -0.449 |
| OTU_1420 | 0.383 | 0.173 | 0.134 | 0.296 | 0.114 | -0.155 | 0.102 | 0.105 | 0.144 | 0.051 | 0.171 | 0.292 | 0.155 | -0.018 | 0.149 | 0.132 | 0.314 |
| OTU_400 | 0.286 | 0.315 | 0.317 | 0.395 | 0.431 | 0.243 | 0.398 | 0.394 | 0.189 | -0.015 | 0.044 | 0.169 | 0.347 | 0.063 | 0.203 | 0.228 | 0.266 |
| OTU_1717 | 0.197 | 0.413 | 0.398 | 0.356 | 0.401 | 0.177 | 0.349 | 0.333 | 0.280 | 0.026 | 0.030 | 0.178 | 0.279 | 0.150 | 0.162 | 0.092 | 0.203 |
| OTU_266 | -0.235 | -0.440 | -0.514 | -0.451 | -0.392 | -0.186 | -0.407 | -0.452 | -0.037 | 0.224 | -0.014 | -0.030 | -0.318 | -0.401 | -0.294 | -0.241 | -0.264 |
| OTU_467 | 0.094 | 0.148 | 0.085 | 0.251 | 0.156 | 0.103 | 0.147 | 0.117 | 0.215 | -0.266 | -0.168 | -0.054 | 0.169 | -0.015 | 0.138 | 0.178 | 0.117 |
| OTU_610 | 0.081 | 0.097 | 0.088 | 0.350 | 0.270 | 0.294 | 0.184 | 0.109 | 0.320 | -0.206 | -0.063 | 0.039 | 0.221 | -0.043 | 0.137 | 0.247 | 0.066 |
| OTU_1581 | 0.159 | 0.194 | 0.163 | 0.335 | 0.432 | 0.198 | 0.429 | 0.249 | 0.120 | -0.149 | -0.176 | -0.108 | 0.063 | 0.020 | 0.237 | 0.140 | 0.190 |
| OTU_423 | 0.086 | 0.154 | 0.267 | 0.092 | -0.034 | -0.018 | -0.013 | 0.004 | -0.160 | -0.129 | 0.002 | -0.054 | -0.030 | 0.355 | -0.211 | -0.305 | -0.140 |
| OTU_264 | 0.259 | 0.188 | 0.153 | 0.437 | 0.350 | 0.144 | 0.344 | 0.251 | 0.192 | -0.172 | -0.100 | 0.068 | 0.244 | 0.009 | 0.406 | 0.325 | 0.345 |
| OTU_488 | 0.391 | 0.260 | 0.277 | 0.352 | 0.239 | 0.027 | 0.228 | 0.268 | 0.079 | 0.120 | 0.267 | 0.217 | 0.189 | 0.083 | 0.082 | -0.072 | 0.289 |
| OTU_335 | 0.332 | 0.209 | 0.210 | 0.388 | 0.360 | 0.264 | 0.320 | 0.227 | 0.239 | -0.001 | -0.004 | 0.175 | 0.204 | 0.004 | 0.120 | 0.197 | 0.321 |
| OTU_759 | 0.321 | 0.247 | 0.234 | 0.417 | 0.316 | 0.160 | 0.291 | 0.257 | 0.073 | -0.088 | 0.043 | 0.007 | 0.212 | 0.000 | 0.086 | 0.027 | 0.356 |
| OTU_631 | 0.288 | 0.245 | 0.189 | 0.347 | 0.224 | -0.059 | 0.239 | 0.134 | -0.058 | 0.052 | 0.057 | 0.013 | 0.074 | 0.094 | 0.239 | 0.221 | 0.131 |
| OTU_451 | 0.258 | 0.276 | 0.270 | 0.356 | 0.281 | 0.167 | 0.223 | 0.288 | 0.189 | 0.358 | 0.099 | 0.059 | 0.194 | 0.189 | 0.083 | -0.053 | 0.184 |
| OTU_845 | 0.255 | 0.345 | 0.351 | 0.463 | 0.490 | 0.353 | 0.411 | 0.365 | 0.377 | 0.169 | 0.100 | 0.256 | 0.260 | 0.064 | -0.024 | 0.019 | 0.241 |
