# Supplementary material for: Roux-en-Y Gastric Bypass Improved Insulin Resistance via Alteration of the Human Gut Microbiome and Alleviation of Endotoxemia
Source: Biomed Res Int. 2021 Jul 12;2021:5554991. doi: 10.1155/2021/5554991 (PMC8294027; doi:10.1155/2021/5554991)
Supplement: Supplementary 5 — Supplemental Table 4. P value of OTU classification and clinical parameters. [file 5554991.f5.docx]

**table 4 *P*-value of OTU classification and clinical parameters**

| **tax** | **BMI** | **ALT** | **AST** | **Gamma GT** | **HOMA**  **-IR** | **FBS** | **Fasting Insulin** | **Fasting c-peptide** | **HbA1c** | **HDL-c** | **LDL-c** | **non-**  **HDL_c** | **Tri-**  **glyceride** | **Uric**  **acid** | **Ghrelin** | **Gastric**  **Inhibitory**  **Polypeptide** | **Leptin** |
| --- | --- | --- | --- | --- | --- | --- | --- | --- | --- | --- | --- | --- | --- | --- | --- | --- | --- |
| OTU_4 | 0.548 | 0.165 | 0.589 | 0.330 | 0.056 | 0.237 | 0.260 | 0.478 | 0.311 | 0.503 | 0.030 | 0.031 | 0.581 | 0.259 | 0.892 | 0.778 | 0.603 |
| OTU_8 | 0.831 | 0.657 | 0.317 | 0.110 | 0.100 | 0.239 | 0.377 | 0.277 | 0.194 | 0.875 | 0.514 | 0.227 | 0.131 | 0.396 | 0.360 | 0.744 | 0.112 |
| OTU_18 | 0.349 | 0.109 | 0.445 | 0.116 | 0.006 | 0.258 | 0.010 | 0.082 | 0.823 | 0.556 | 0.773 | 0.799 | 0.165 | 0.525 | 0.314 | 0.515 | 0.084 |
| OTU_28 | 0.019 | 0.001 | 0.002 | 0.001 | 0.003 | 0.028 | 0.010 | 0.039 | 0.061 | 0.668 | 0.462 | 0.934 | 0.071 | 0.148 | 0.070 | 0.205 | 0.054 |
| OTU_29 | 0.137 | 0.003 | 0.001 | 0.001 | 0.014 | 0.043 | 0.067 | 0.033 | 0.215 | 0.674 | 0.169 | 0.041 | 0.119 | 0.809 | 0.171 | 0.256 | 0.008 |
| OTU_129 | 0.450 | 0.646 | 0.368 | 0.539 | 0.197 | 0.714 | 0.087 | 0.122 | 0.258 | 0.842 | 0.849 | 0.819 | 0.619 | 0.897 | 0.983 | 0.991 | 0.126 |
| OTU_302 | 0.010 | 0.013 | 0.025 | 0.001 | 0.008 | 0.145 | 0.064 | 0.261 | 0.347 | 0.444 | 0.753 | 0.838 | 0.229 | 0.041 | 0.021 | 0.037 | 0.056 |
| OTU_83 | 0.336 | 0.623 | 0.735 | 0.651 | 0.629 | 0.219 | 0.280 | 0.184 | 0.065 | 0.962 | 0.658 | 0.588 | 0.627 | 0.028 | 0.676 | 0.324 | 0.095 |
| OTU_115 | 0.064 | 0.922 | 0.983 | 0.008 | 0.001 | 0.475 | 0.001 | 0.002 | 0.144 | 0.687 | 0.173 | 0.438 | 0.281 | 0.698 | 0.797 | 0.623 | 0.001 |
| OTU_116 | 0.102 | 0.496 | 0.527 | 0.175 | 0.115 | 0.560 | 0.232 | 0.477 | 0.739 | 0.136 | 0.705 | 0.312 | 0.400 | 0.278 | 0.069 | 0.751 | 0.119 |
| OTU_184 | 0.725 | 0.847 | 0.522 | 0.021 | 0.074 | 0.005 | 0.571 | 0.307 | 0.002 | 0.195 | 0.074 | 0.010 | 0.061 | 0.115 | 0.003 | 0.051 | 0.036 |
| OTU_136 | 0.036 | 0.057 | 0.083 | 0.002 | 0.002 | 0.007 | 0.009 | 0.005 | 0.043 | 0.251 | 0.963 | 0.346 | 0.089 | 0.423 | 0.163 | 0.167 | 0.001 |
| OTU_114 | 0.165 | 0.215 | 0.317 | 0.014 | 0.091 | 0.599 | 0.069 | 0.041 | 0.295 | 0.889 | 0.308 | 0.143 | 0.316 | 0.665 | 0.186 | 0.361 | 0.007 |
| OTU_171 | 0.471 | 0.169 | 0.453 | 0.033 | 0.186 | 0.397 | 0.212 | 0.161 | 0.279 | 0.599 | 0.364 | 0.086 | 0.052 | 0.261 | 0.182 | 0.478 | 0.185 |
| OTU_756 | 0.220 | 0.013 | 0.003 | 0.001 | 0.004 | 0.016 | 0.030 | 0.006 | 0.091 | 0.684 | 0.183 | 0.016 | 0.008 | 0.934 | 0.060 | 0.107 | 0.014 |
| OTU_138 | 0.119 | 0.579 | 0.944 | 0.115 | 0.123 | 0.169 | 0.266 | 0.403 | 0.419 | 0.342 | 0.937 | 0.408 | 0.526 | 0.806 | 0.006 | 0.008 | 0.018 |
| OTU_247 | 0.038 | 0.154 | 0.073 | 0.001 | 0.137 | 0.668 | 0.089 | 0.202 | 0.848 | 0.094 | 0.882 | 0.741 | 0.308 | 0.080 | 0.704 | 0.314 | 0.089 |
| OTU_212 | 0.039 | 0.254 | 0.072 | 0.003 | 0.005 | 0.521 | 0.002 | 0.018 | 0.770 | 0.010 | 0.412 | 0.491 | 0.177 | 0.428 | 0.065 | 0.146 | 0.007 |
| OTU_170 | 0.401 | 0.601 | 0.454 | 0.317 | 0.413 | 0.907 | 0.311 | 0.257 | 0.947 | 0.378 | 0.351 | 0.493 | 0.732 | 0.113 | 0.038 | 0.098 | 0.065 |
| OTU_1452 | 0.140 | 0.131 | 0.109 | 0.015 | 0.019 | 0.774 | 0.007 | 0.084 | 0.762 | 0.560 | 0.833 | 0.917 | 0.786 | 0.449 | 0.232 | 0.734 | 0.126 |
| OTU_1005 | 0.001 | 0.042 | 0.007 | 0.001 | 0.010 | 0.219 | 0.004 | 0.011 | 0.049 | 0.431 | 0.201 | 0.337 | 0.072 | 0.210 | 0.045 | 0.005 | 0.004 |
| OTU_906 | 0.002 | 0.130 | 0.034 | 0.001 | 0.033 | 0.124 | 0.037 | 0.098 | 0.711 | 0.249 | 0.626 | 0.917 | 0.119 | 0.593 | 0.077 | 0.020 | 0.007 |
| OTU_263 | 0.190 | 0.001 | 0.001 | 0.005 | 0.033 | 0.378 | 0.061 | 0.015 | 0.363 | 0.225 | 0.850 | 0.939 | 0.021 | 0.109 | 0.004 | 0.060 | 0.228 |
| OTU_433 | 0.200 | 0.503 | 0.641 | 0.050 | 0.353 | 0.850 | 0.126 | 0.254 | 0.939 | 0.570 | 0.917 | 0.773 | 0.804 | 0.452 | 0.498 | 0.747 | 0.025 |
| OTU_236 | 0.118 | 0.636 | 0.911 | 0.014 | 0.002 | 0.004 | 0.018 | 0.242 | 0.008 | 0.626 | 0.330 | 0.543 | 0.807 | 0.987 | 0.782 | 0.832 | 0.043 |
| OTU_259 | 0.045 | 0.028 | 0.012 | 0.002 | 0.001 | 0.031 | 0.005 | 0.001 | 0.027 | 0.439 | 0.939 | 0.260 | 0.029 | 0.139 | 0.848 | 0.961 | 0.039 |
| OTU_235 | 0.011 | 0.035 | 0.022 | 0.001 | 0.012 | 0.131 | 0.038 | 0.001 | 0.019 | 0.314 | 0.045 | 0.039 | 0.002 | 0.364 | 0.045 | 0.051 | 0.002 |
| OTU_413 | 0.597 | 0.061 | 0.159 | 0.582 | 0.707 | 0.552 | 0.706 | 0.810 | 0.532 | 0.647 | 0.385 | 0.125 | 0.384 | 0.188 | 0.920 | 0.449 | 0.642 |
| OTU_510 | 0.033 | 0.086 | 0.057 | 0.001 | 0.001 | 0.019 | 0.025 | 0.010 | 0.004 | 0.384 | 0.506 | 0.072 | 0.022 | 0.210 | 0.832 | 0.817 | 0.007 |
| OTU_1256 | 0.017 | 0.029 | 0.013 | 0.001 | 0.003 | 0.082 | 0.016 | 0.002 | 0.226 | 0.143 | 0.561 | 0.434 | 0.003 | 0.322 | 0.032 | 0.046 | 0.002 |
| OTU_303 | 0.563 | 0.158 | 0.833 | 0.130 | 0.048 | 0.094 | 0.090 | 0.027 | 0.055 | 0.984 | 0.391 | 0.182 | 0.020 | 0.570 | 0.437 | 0.082 | 0.019 |
| OTU_499 | 0.099 | 0.004 | 0.002 | 0.001 | 0.001 | 0.008 | 0.008 | 0.004 | 0.136 | 0.677 | 0.575 | 0.161 | 0.027 | 0.963 | 0.030 | 0.023 | 0.014 |
| OTU_4535 | 0.074 | 0.286 | 0.367 | 0.091 | 0.050 | 0.176 | 0.075 | 0.010 | 0.024 | 0.680 | 0.851 | 0.668 | 0.095 | 0.535 | 0.898 | 0.458 | 0.003 |
| OTU_1420 | 0.002 | 0.118 | 0.059 | 0.003 | 0.160 | 0.136 | 0.566 | 0.577 | 0.058 | 0.704 | 0.167 | 0.005 | 0.189 | 0.641 | 0.071 | 0.053 | 0.168 |
| OTU_400 | 0.024 | 0.002 | 0.002 | 0.001 | 0.001 | 0.023 | 0.035 | 0.019 | 0.090 | 0.763 | 0.327 | 0.052 | 0.035 | 0.924 | 0.031 | 0.015 | 0.248 |
| OTU_1717 | 0.180 | 0.001 | 0.001 | 0.001 | 0.003 | 0.010 | 0.050 | 0.052 | 0.041 | 0.802 | 0.411 | 0.106 | 0.034 | 0.647 | 0.798 | 0.761 | 0.777 |
| OTU_266 | 0.038 | 0.037 | 0.045 | 0.026 | 0.014 | 0.553 | 0.014 | 0.003 | 0.721 | 0.176 | 0.541 | 0.557 | 0.082 | 0.002 | 0.095 | 0.259 | 0.091 |
| OTU_467 | 0.505 | 0.964 | 0.863 | 0.738 | 0.049 | 0.012 | 0.730 | 0.322 | 0.003 | 0.063 | 0.292 | 0.938 | 0.021 | 0.693 | 0.485 | 0.912 | 0.952 |
| OTU_610 | 0.512 | 0.114 | 0.122 | 0.001 | 0.073 | 0.052 | 0.199 | 0.570 | 0.021 | 0.433 | 0.565 | 0.878 | 0.358 | 0.595 | 0.161 | 0.134 | 0.274 |
| OTU_1581 | 0.097 | 0.620 | 0.876 | 0.006 | 0.001 | 0.019 | 0.002 | 0.053 | 0.073 | 0.554 | 0.224 | 0.212 | 0.496 | 0.392 | 0.335 | 0.840 | 0.055 |
| OTU_423 | 0.737 | 0.623 | 0.468 | 0.402 | 0.928 | 0.496 | 0.897 | 0.886 | 0.250 | 0.234 | 0.896 | 0.990 | 0.873 | 0.010 | 0.164 | 0.184 | 0.203 |
| OTU_264 | 0.025 | 0.316 | 0.636 | 0.001 | 0.001 | 0.020 | 0.012 | 0.050 | 0.012 | 0.253 | 0.454 | 0.759 | 0.001 | 0.873 | 0.003 | 0.001 | 0.152 |
| OTU_488 | 0.018 | 0.078 | 0.051 | 0.001 | 0.012 | 0.143 | 0.041 | 0.021 | 0.153 | 0.307 | 0.107 | 0.113 | 0.113 | 0.753 | 0.153 | 0.183 | 0.011 |
| OTU_335 | 0.019 | 0.050 | 0.023 | 0.007 | 0.004 | 0.003 | 0.147 | 0.303 | 0.012 | 0.947 | 0.980 | 0.207 | 0.067 | 0.647 | 0.124 | 0.011 | 0.006 |
| OTU_759 | 0.032 | 0.082 | 0.032 | 0.025 | 0.013 | 0.082 | 0.137 | 0.135 | 0.119 | 0.490 | 0.637 | 0.980 | 0.178 | 0.836 | 0.069 | 0.064 | 0.001 |
| OTU_631 | 0.036 | 0.024 | 0.050 | 0.001 | 0.045 | 0.143 | 0.129 | 0.436 | 0.181 | 0.639 | 0.930 | 0.952 | 0.593 | 0.828 | 0.001 | 0.004 | 0.378 |
| OTU_451 | 0.097 | 0.065 | 0.031 | 0.003 | 0.003 | 0.066 | 0.023 | 0.014 | 0.042 | 0.008 | 0.693 | 0.660 | 0.046 | 0.469 | 0.621 | 0.790 | 0.015 |
| OTU_845 | 0.042 | 0.008 | 0.002 | 0.002 | 0.001 | 0.005 | 0.008 | 0.005 | 0.007 | 0.070 | 0.623 | 0.042 | 0.027 | 0.657 | 0.579 | 0.934 | 0.037 |
