# Supplementary material for: Roux-en-Y Gastric Bypass Improved Insulin Resistance via Alteration of the Human Gut Microbiome and Alleviation of Endotoxemia
Source: Biomed Res Int. 2021 Jul 12;2021:5554991. doi: 10.1155/2021/5554991 (PMC8294027; doi:10.1155/2021/5554991)
Supplement: Supplementary 6 — Supplemental Table 5. Correlation between OTU classification and serum LBP concentration. [file 5554991.f6.docx]

**table 5 Correlation between OTU classification and serum LBP concentration.**

| OTU | Env | Correlation | *P*-value |
| --- | --- | --- | --- |
| OTU_18 | LBP | -0.304 | 0.036 |
| OTU_302 | LBP | -0.172 | 0.012 |
| OTU_115 | LBP | -0.226 | 0.039 |
| OTU_247 | LBP | 0.342 | 0.024 |
| OTU_212 | LBP | 0.354 | 0.026 |
| OTU_1452 | LBP | 0.558 | 0.007 |
| OTU_1005 | LBP | 0.401 | 0.008 |
| OTU_906 | LBP | 0.358 | 0.012 |
| OTU_236 | LBP | 0.333 | 0.023 |
| OTU_259 | LBP | 0.337 | 0.015 |
| OTU_4535 | LBP | -0.320 | 0.008 |
| OTU_335 | LBP | 0.337 | 0.017 |
