# Supplementary material for: Roux-en-Y Gastric Bypass Improved Insulin Resistance via Alteration of the Human Gut Microbiome and Alleviation of Endotoxemia
Source: Biomed Res Int. 2021 Jul 12;2021:5554991. doi: 10.1155/2021/5554991 (PMC8294027; doi:10.1155/2021/5554991)
Supplement: Supplementary 7 — Supplemental Table 6. Correlation between OTU classification and feaces SCFAs concentration. [file 5554991.f7.docx]

**table 6 Correlation between OTU classification and feaces SCFAs concentration.**

| OTUID | acetate | butyrate | pentanoate | propionate | isopentanoate | isobutyrate |
| --- | --- | --- | --- | --- | --- | --- |
| OTU_4 | -0.107 | -0.048 | 0.127 | -0.081 | -0.101 | -0.078 |
| OTU_8 | 0.172 | 0.280 | 0.323 | 0.276 | 0.340 | 0.309 |
| OTU_18 | 0.146 | 0.352 | 0.274 | 0.184 | 0.384 | 0.376 |
| OTU_28 | 0.457 | 0.577 | 0.358 | 0.425 | 0.398 | 0.394 |
| OTU_29 | -0.251 | -0.252 | -0.348 | -0.336 | -0.230 | -0.196 |
| OTU_129 | -0.351 | -0.393 | -0.070 | -0.345 | -0.306 | -0.262 |
| OTU_302 | 0.528 | 0.717 | 0.411 | 0.501 | 0.476 | 0.479 |
| OTU_83 | -0.037 | 0.140 | 0.154 | 0.098 | -0.041 | -0.049 |
| OTU_115 | 0.098 | 0.070 | 0.031 | 0.039 | 0.255 | 0.240 |
| OTU_116 | -0.152 | -0.221 | 0.114 | -0.210 | 0.300 | 0.288 |
| OTU_184 | 0.517 | 0.593 | 0.373 | 0.589 | 0.658 | 0.629 |
| OTU_136 | 0.485 | 0.607 | 0.473 | 0.524 | 0.546 | 0.522 |
| OTU_114 | -0.345 | -0.386 | -0.178 | -0.352 | -0.366 | -0.325 |
| OTU_171 | 0.483 | 0.528 | 0.350 | 0.526 | 0.424 | 0.368 |
| OTU_756 | -0.233 | -0.290 | -0.399 | -0.397 | -0.295 | -0.274 |
| OTU_138 | -0.144 | -0.234 | -0.313 | -0.210 | -0.316 | -0.310 |
| OTU_247 | -0.037 | -0.057 | -0.252 | -0.165 | -0.245 | -0.198 |
| OTU_212 | -0.169 | -0.283 | -0.430 | -0.420 | -0.388 | -0.364 |
| OTU_170 | 0.064 | 0.220 | 0.294 | 0.116 | 0.294 | 0.257 |
| OTU_1452 | -0.125 | -0.211 | -0.239 | -0.119 | -0.208 | -0.222 |
| OTU_1005 | -0.226 | -0.227 | -0.308 | -0.314 | -0.329 | -0.336 |
| OTU_906 | -0.075 | -0.123 | -0.371 | -0.157 | -0.259 | -0.200 |
| OTU_263 | -0.275 | -0.419 | -0.437 | -0.386 | -0.288 | -0.264 |
| OTU_433 | -0.522 | -0.508 | -0.215 | -0.505 | -0.431 | -0.418 |
| OTU_236 | -0.324 | -0.310 | -0.129 | -0.314 | -0.312 | -0.294 |
| OTU_259 | -0.263 | -0.247 | -0.325 | -0.278 | -0.256 | -0.199 |
| OTU_235 | -0.243 | -0.350 | -0.496 | -0.432 | -0.359 | -0.316 |
| OTU_413 | -0.001 | 0.042 | 0.283 | 0.047 | 0.072 | 0.027 |
| OTU_510 | -0.170 | -0.152 | -0.332 | -0.247 | -0.267 | -0.199 |
| OTU_1256 | -0.176 | -0.237 | -0.468 | -0.372 | -0.353 | -0.323 |
| OTU_303 | 0.213 | 0.395 | 0.295 | 0.368 | 0.260 | 0.225 |
| OTU_499 | -0.277 | -0.270 | -0.329 | -0.303 | -0.283 | -0.258 |
| OTU_4535 | 0.171 | 0.184 | 0.364 | 0.251 | 0.324 | 0.291 |
| OTU_1420 | -0.171 | -0.159 | -0.231 | -0.180 | -0.315 | -0.308 |
| OTU_400 | -0.263 | -0.277 | -0.299 | -0.313 | -0.300 | -0.249 |
| OTU_1717 | -0.264 | -0.311 | -0.278 | -0.308 | -0.295 | -0.297 |
| OTU_266 | 0.113 | 0.298 | 0.285 | 0.216 | 0.020 | -0.006 |
| OTU_467 | -0.169 | -0.155 | -0.161 | -0.199 | -0.377 | -0.374 |
| OTU_610 | -0.310 | -0.368 | -0.220 | -0.294 | -0.371 | -0.387 |
| OTU_1581 | -0.361 | -0.358 | -0.202 | -0.330 | -0.406 | -0.394 |
| OTU_423 | 0.128 | 0.120 | 0.083 | 0.146 | 0.357 | 0.314 |
| OTU_264 | -0.400 | -0.439 | -0.370 | -0.453 | -0.519 | -0.502 |
| OTU_488 | -0.183 | -0.169 | -0.294 | -0.290 | -0.347 | -0.352 |
| OTU_335 | -0.229 | -0.234 | -0.276 | -0.214 | -0.371 | -0.371 |
| OTU_759 | -0.317 | -0.284 | -0.270 | -0.329 | -0.292 | -0.295 |
| OTU_631 | -0.444 | -0.372 | -0.193 | -0.426 | -0.375 | -0.379 |
| OTU_451 | -0.297 | -0.344 | -0.325 | -0.468 | -0.395 | -0.413 |
| OTU_845 | -0.320 | -0.330 | -0.316 | -0.435 | -0.362 | -0.364 |
