# Supplementary material for: Roux-en-Y Gastric Bypass Improved Insulin Resistance via Alteration of the Human Gut Microbiome and Alleviation of Endotoxemia
Source: Biomed Res Int. 2021 Jul 12;2021:5554991. doi: 10.1155/2021/5554991 (PMC8294027; doi:10.1155/2021/5554991)
Supplement: Supplementary 8 — Supplemental Table 7. P-value of OTU classification and feaces SCFAs concentration. [file 5554991.f8.docx]

**table 7 *P*-value of OTU classification and feaces SFCAs concentration.**

| **OTUID** | **acetate** | **butyrate** | **pentanoate** | **propionate** | **isopentanoate** | **isobutyrate** |
| --- | --- | --- | --- | --- | --- | --- |
| OTU_4 | 0.067 | 0.052 | 0.213 | 0.244 | 0.177 | 0.132 |
| OTU_8 | 0.081 | 0.006 | 0.017 | 0.002 | 0.010 | 0.030 |
| OTU_18 | 0.005 | 0.001 | 0.068 | 0.031 | 0.003 | 0.001 |
| OTU_28 | 0.003 | 0.001 | 0.021 | 0.001 | 0.002 | 0.001 |
| OTU_29 | 0.194 | 0.201 | 0.027 | 0.030 | 0.135 | 0.194 |
| OTU_129 | 0.036 | 0.034 | 0.075 | 0.079 | 0.105 | 0.205 |
| OTU_302 | 0.001 | 0.001 | 0.016 | 0.004 | 0.003 | 0.002 |
| OTU_83 | 0.443 | 0.672 | 0.725 | 0.836 | 0.772 | 0.689 |
| OTU_115 | 0.617 | 0.300 | 0.485 | 0.551 | 0.134 | 0.187 |
| OTU_116 | 0.600 | 0.326 | 0.040 | 0.142 | 0.011 | 0.016 |
| OTU_184 | 0.001 | 0.001 | 0.006 | 0.001 | 0.001 | 0.002 |
| OTU_136 | 0.001 | 0.002 | 0.002 | 0.003 | 0.002 | 0.002 |
| OTU_114 | 0.010 | 0.003 | 0.081 | 0.010 | 0.025 | 0.057 |
| OTU_171 | 0.006 | 0.002 | 0.021 | 0.001 | 0.004 | 0.020 |
| OTU_756 | 0.162 | 0.050 | 0.019 | 0.004 | 0.036 | 0.071 |
| OTU_138 | 0.561 | 0.126 | 0.069 | 0.449 | 0.082 | 0.112 |
| OTU_247 | 0.117 | 0.111 | 0.143 | 0.244 | 0.153 | 0.269 |
| OTU_212 | 0.275 | 0.063 | 0.033 | 0.003 | 0.002 | 0.009 |
| OTU_170 | 0.195 | 0.026 | 0.010 | 0.610 | 0.051 | 0.055 |
| OTU_1452 | 0.646 | 0.355 | 0.258 | 0.595 | 0.359 | 0.361 |
| OTU_1005 | 0.176 | 0.238 | 0.140 | 0.110 | 0.084 | 0.097 |
| OTU_906 | 0.790 | 0.638 | 0.013 | 0.437 | 0.116 | 0.223 |
| OTU_263 | 0.081 | 0.005 | 0.016 | 0.056 | 0.138 | 0.146 |
| OTU_433 | 0.001 | 0.003 | 0.132 | 0.001 | 0.002 | 0.002 |
| OTU_236 | 0.057 | 0.107 | 0.274 | 0.088 | 0.060 | 0.063 |
| OTU_259 | 0.098 | 0.153 | 0.072 | 0.081 | 0.168 | 0.281 |
| OTU_235 | 0.092 | 0.018 | 0.003 | 0.009 | 0.011 | 0.030 |
| OTU_413 | 0.968 | 0.750 | 0.011 | 0.909 | 0.344 | 0.565 |
| OTU_510 | 0.186 | 0.445 | 0.031 | 0.137 | 0.146 | 0.221 |
| OTU_1256 | 0.352 | 0.215 | 0.004 | 0.027 | 0.021 | 0.046 |
| OTU_303 | 0.089 | 0.051 | 0.240 | 0.008 | 0.163 | 0.236 |
| OTU_499 | 0.072 | 0.085 | 0.026 | 0.028 | 0.025 | 0.062 |
| OTU_4535 | 0.378 | 0.166 | 0.008 | 0.303 | 0.020 | 0.048 |
| OTU_1420 | 0.286 | 0.468 | 0.040 | 0.393 | 0.056 | 0.071 |
| OTU_400 | 0.038 | 0.074 | 0.039 | 0.035 | 0.042 | 0.084 |
| OTU_1717 | 0.123 | 0.074 | 0.032 | 0.043 | 0.036 | 0.041 |
| OTU_266 | 0.784 | 0.211 | 0.139 | 0.189 | 0.789 | 0.813 |
| OTU_467 | 0.427 | 0.387 | 0.436 | 0.583 | 0.025 | 0.033 |
| OTU_610 | 0.030 | 0.028 | 0.256 | 0.047 | 0.038 | 0.025 |
| OTU_1581 | 0.035 | 0.022 | 0.206 | 0.176 | 0.028 | 0.039 |
| OTU_423 | 0.109 | 0.400 | 0.747 | 0.220 | 0.022 | 0.035 |
| OTU_264 | 0.008 | 0.007 | 0.009 | 0.005 | 0.002 | 0.005 |
| OTU_488 | 0.337 | 0.393 | 0.213 | 0.235 | 0.265 | 0.246 |
| OTU_335 | 0.114 | 0.175 | 0.088 | 0.177 | 0.018 | 0.019 |
| OTU_759 | 0.028 | 0.091 | 0.062 | 0.050 | 0.072 | 0.080 |
| OTU_631 | 0.003 | 0.018 | 0.099 | 0.006 | 0.028 | 0.015 |
| OTU_451 | 0.046 | 0.029 | 0.047 | 0.005 | 0.019 | 0.015 |
| OTU_845 | 0.044 | 0.055 | 0.043 | 0.007 | 0.017 | 0.030 |
