# Supplementary material for: Roux-en-Y Gastric Bypass Improved Insulin Resistance via Alteration of the Human Gut Microbiome and Alleviation of Endotoxemia
Source: Biomed Res Int. 2021 Jul 12;2021:5554991. doi: 10.1155/2021/5554991 (PMC8294027; doi:10.1155/2021/5554991)
Supplement: Supplementary 9 — Supplemental Table 8. Correlation between OTU classification and serum TMAO levels. [file 5554991.f9.docx]

**table 8 Correlation between OTU classification and serum TMAO levels.**

| **OTU** | **Env** | **Correlation** | *P*-value |
| --- | --- | --- | --- |
| OTU_302 | TMAO | 0.421 | 0.012 |
| OTU_756 | TMAO | -0.102 | 0.043 |
| OTU_1256 | TMAO | -0.025 | 0.016 |
| OTU_610 | TMAO | -0.359 | 0.014 |
| OTU_264 | TMAO | -0.415 | 0.045 |
| OTU_631 | TMAO | -0.412 | 0.050 |
